# Outcomes in elderly patients admitted to the intensive care unit with solid tumors

**DOI:** 10.1186/s13613-017-0250-0

**Published:** 2017-03-06

**Authors:** Edouard Auclin, Anaïs Charles-Nelson, Baptiste Abbar, Emmanuel Guérot, Stéphane Oudard, Caroline Hauw-Berlemont, Constance Thibault, Alexandra Monnier, Jean-Luc Diehl, Sandrine Katsahian, Jean-Yves Fagon, Julien Taieb, Nadia Aissaoui

**Affiliations:** 1 Gastrointestinal Oncology Department, European Georges Pompidou Hospital, Paris, France; 2 Intensive Care Unit, European Georges Pompidou Hospital, Paris, France; 3 Oncology Department, European Georges Pompidou Hospital, Paris, France; 4 Université Paris Descartes, Paris, France; 5 Clinical Research Unit, European Georges Pompidou Hospital, Paris, France

**Keywords:** Elderly, Cancer, Outcome, Intensive care, Survival, Treatment

## Abstract

**Background:**

As the population ages and cancer therapies improve, there is an increased call for elderly cancer patients to be admitted to the intensive care unit (ICU). This study aimed to assess short-term survival and prognostic factors in critically ill patients with solid tumors aged ≥65 years.

**Methods:**

We conducted a retrospective study. The primary endpoint was ICU mortality. Resumption of anticancer therapy in patients who survived the ICU stay and 90-day mortality were secondary endpoints. All patients aged ≥65 years admitted to the ICU of Georges Pompidou Hospital (Paris, France) between 2009 and 2014 were eligible.

**Results:**

Of 2327 eligible elderly patients (EP), 262 (75.0 ± 6.7 years) with solid tumors were analyzed. These patients were extremely critically ill (SAPS 2 61.9 ± 22.5), and 60.3% had metastatic disease. Gastrointestinal, lung and genitourinary cancers were the most common types of tumors. Mechanical ventilation was required in 51.5% of patients, inotropes in 48.1% and dialysis in 12.6%. Most patients (66.7%) were admitted for reasons unrelated to cancer, including sepsis (30.5%), acute respiratory failure (28.2%) and neurological problems (8.0%). ICU mortality in patients with cancer was 33.6 versus 32.6% among patients without cancer (*p* = 0.75). Among the cancer EP, the 90-day mortality was 51.9% (*n* = 136). In multivariate analysis, increased SAPS 2 score and primary tumor site were associated with 90-day death, whereas previous anticancer therapies and poor performance status were not. Among survivor patients from ICU with anti-tumoral treatment indication, 77 (52.7%) had resumption of anticancer treatment.

**Conclusions:**

Elderly solid tumor patients admitted to the ICU had a mortality rate similar to EP without cancer. Prognostic factors for 90-day mortality were more related to severity of clinical status at admission than the presence or stage of cancer, suggesting that early admission of EP with cancer to the ICU is appropriate.

**Electronic supplementary material:**

The online version of this article (doi:10.1186/s13613-017-0250-0) contains supplementary material, which is available to authorized users.

## Background

Cancer is the leading cause of mortality in France, ahead of cardiovascular diseases. In 2015 there were 385,000 new cases of cancer in France, with 149,500 cancer-related deaths [1]. The median age at diagnosis of cancer is 65 years, and the rate of cancer diagnosis increases with age in both males and females [2].

Remarkable advances have been made in the early diagnosis and management of patients with malignancies, resulting in dramatic improvements in overall survival rates [3, 4]. As a result, increasing numbers of oncology patients are admitted to the intensive care unit (ICU), for either life-threatening cancer-related complications, treatment-associated side effects or standard critical care admission indications [5].

Overall, survival rates in critically ill patients with active cancer appear to be increasing [5]. However, studies conducted among patients with hematological malignancies or lung cancer indicate that ICU mortality in these patients is high [6–8]. In addition, a number of unanswered questions remain, including a lack of follow-up data on patients who survive a stay in ICU, and data on the clinical course and anticancer treatment continuation rates after the critical illness are very limited [8, 9]. Furthermore, there is a lack of data on ICU patients with non-hematological malignancies.

The aim of this study was to assess patient outcomes and identify factors associated with 90-day mortality and antineoplastic treatment resumption in elderly patients with metastatic or non-metastatic solid cancers admitted to the ICU.

## Methods

### Study design

This retrospective observational study was conducted between 2009 and 2014 in the ICU of the European Georges Pompidou Hospital (Paris, France). The protocol was approved by the local institutional ethics committee, and the study was performed in accordance with the ethical standards laid down in the 1964 Declaration of Helsinki.

### Patients

Consecutive patients were eligible for inclusion if they were aged 65 years or older, with or without a diagnosis of malignant solid tumor. Patients with hematological malignancy, an ICU stay of <24 h with limitation of active therapies, cancer diagnosed during the ICU stay, or cancer remission of more than 5 years were excluded.

### Data collection

Data were collected retrospectively from patients’ computerized medical files using the Dxcare software. Demographic data, performance status, cancer data (primary tumor, presence of metastases, metastatic sites, number of previous anticancer treatments, type of treatment and neutropenia status), reasons for ICU admission, status at admission assessed using the SAPS 2 score [10], life-support therapies used and the duration of use (mechanical ventilation, inotropes), and biological data at admission were collected. Reasons for admission were classified in two ways: one based on relationship with cancer (related to cancer progression or antineoplastic treatment toxicity, or no link with cancer) and the second based on medical diagnosis at admission (sepsis, acute heart failure, hemorrhagic shock, hypovolemia, acute respiratory failure, chronic bronchitis exacerbation, neurological reasons (i.e., coma and/or seizure), acute kidney failure, electrolytic disorders, cardiorespiratory shock, post-surgery, medical surveillance, unknown shock). The reason for admission was considered as the symptom at the origin of the ICU request.

### Outcomes

ICU mortality rates were determined for elderly patients with and without solid tumors. For those with solid tumors, in-hospital mortality, 90-day mortality and anticancer treatment resumption were also assessed.

### Statistical analysis

Statistical analysis was performed using R software. For quantitative variables, mean and standard deviations were calculated. Discrete variables are presented as percentages. Comparisons between patient characteristics were performed using Chi-square or Fisher’s exact test for discrete variables and using the unpaired t test, Wilcoxon sign-rank test or analysis of variance for continuous variables.

Predictive factors for 90-day mortality were tested in univariate and multivariate analyses using a Cox proportional hazards model. The proportional hazards assumption was verified using the Schoenfeld residuals method. Variables included in the final multivariable models were selected according to their physiological relevance and statistical significance in univariate analysis, using a *p* value threshold of 0.10. Two multivariable analyses were conducted among cancer patients: the first included only variables available on admission: age, comorbidity, performance status, cancer type, metastatic status, SAPS 2 and biological data. The second model added in-ICU management variables (respiratory, circulatory or renal support techniques). Analyses were repeated using forward stepwise analysis to assess the consistency of results. Collinearity was assessed by calculating variance inflation factors. In addition, logistic regression was used to assess factors predictive of definitive anticancer treatment cessation, and the final model was defined using a stepwise method. For all analyses, a *p* value <0.05 was considered statistically significant.

## Results

**Fig. 1:**
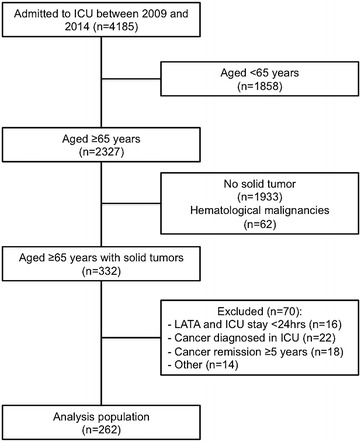
Flow of patients through the study. *ICU* intensive care unit, *LATA* limitation of active therapies

A total of 4185 patients were admitted to the ICU over the study period. Of these, 2327 were classified as elderly (age ≥65 years), 332 (14.3%) of whom had a solid tumor, and 262 (11.3%) were included in the final analysis (Fig. 1).

### Elderly patients with or without solid tumor

The mean age ± standard deviation of all elderly patients (*n* = 2327) was 77.1 ± 8.1 years (1311 male; 56.3%). Patients with cancer were significantly younger than those without cancer (75.2 ± 6.7 vs 79.0 ± 8.2 years; *p* < 0.0001). The mean ± standard deviation IGS2 score in all elderly patients was 59.0 ± 23.0. The SAPS 2 score on admission was significantly higher in cancer versus non-cancer patients (61.9 ± 22.5 vs 56.9 ± 22.4; *p* < 0.0001), as was the McCabe score (2.1 ± 0.6 vs 1.3 ± 0.6; *p* < 0.025). Further details of patient characteristics within the overall population are shown in Additional file 1: Table S1.

The ICU mortality for all elderly patients was 32.5% and did not differ between those with and without solid tumor (33.6 vs 32.7%; *p* = 0.78). Cancer was not associated with in-ICU survival (OR for patients without cancer 0.96; 95% CI 0.73–1.26; *p* = 0.78).

### Elderly patients with solid tumor

#### Characteristics

Full details of demographic and clinical characteristics for patients with solid tumor are reported in Table 1, overall and based on survivorship. Gastrointestinal, lung and genitourinary cancers were the most common types of tumors (Table 1). Sixty-three percent of patients had received at least one previous line of systemic anti-tumoral treatment (Table 1). Sepsis and acute respiratory failure were the two most common reasons for ICU admission (Table 2). Based on SAPS 2 score, patients were critically ill at admission and 135 (51.5%) required mechanical ventilation (Table 2). Median ICU stay was 4.0 days (interquartile range 2.0–7.0). Laboratory parameters for the 262 elderly patients with solid tumors are shown in Additional file 1: Table S2.

**Table 1:** Baseline patient demographics and clinical characteristics for the overall study population and by survivor status

	Overall (*n* = 262)	ICU survivors (*n* = 174)	ICU non-survivors (*n* = 88)	*p* value
Age (years)	75.2 ± 6.7	75.7 ± 6.8	74.3 ± 6.5	0.13
Male	162 (61.8)	101 (58.0)	61 (69.3)	0.08
Comorbidities				
Tobacco use	106 (40.5)	65 (37.4)	41 (46.6)	0.15
Diabetes	57 (21.8)	35 (20.1)	22 (25)	0.36
Chronic respiratory failure	33 (12.6)	23 (13.2)	10 (11.4)	0.18
Chronic kidney failure	29 (11.1)	23 (13.2)	6 (6.8)	0.12
Primary tumor site				0.02
Gastrointestinal	71 (27.1)	52 (29.9)	19 (21.6)	
Lung	68 (26)	45 (25.9)	23 (26.1)	
Genitourinary	60 (22.9)	43 (24.7)	17 (19.3)	
Head and neck	31 (11.8)	11 (6.3)	20 (22.7)	
Breast	18 (6.9)	13 (7.5)	5 (5.7)	
Gynecological	9 (3.4)	7 (4)	2 (2.3)	
Sarcoma	3 (1.1)	2 (1.2)	1 (1.1)	
Unknown	2 (0.8)	1 (0.6)	1 (1.1)	
Metastasis				
Yes	158 (60.3)	110 (63.2)	48 (54.6)	0.17
Other	66 (25.3)	42 (24.3)	24 (27.3)	0.58
Lymph node	58 (22.2)	39 (22.5)	19 (21.6)	0.93
Lung	57 (21.8)	41 (23.7)	16 (18.2)	0.32
Bone	46 (17.6)	31 (17.9)	15 (17.1)	0.88
Liver	38 (14.6)	26 (15)	12 (13.6)	0.78
Brain	9 (3.5)	6 (3.5)	3 (3.4)	0.73
Pulmonary lymphangitis	6 (2.3)	4 (2.3)	2 (2.3)	0.67
Missing	1 (0.4)	1 (0.6)	0 (0)	
Anticancer treatment before ICU admission				
Number of previous systemic anti-tumoral treatments [median (IQR)]	1 (0;1)	1 (0;1)	1 (0;1)	0.32
Missing	8 (3)	5 (2.9)	3 (3.4)	
Chemotherapy	94 (56.3)	66 (60.6)	28 (48.3)	0.18
Targeted therapy	31 (18.7)	22 (20.2)	9 (15.8)	
Missing	95 (36.3)	65 (37.3)	30 (34)	
Performance status				
0 or 1	98 (46.4)	64 (45.7)	34 (47.9)	0.98
2	86 (40.8)	58 (41.4)	28 (39.4)	
3 or 4	27 (12.9)	18 (12.8)	9 (12.7)	
Missing	51 (19.5)	34 (19.5)	17 (19.3)	

Data are mean ± SD or number of patients (%)

**Table 2:** Baseline patient ICU details for the overall study population and by survivor status

	Overall (*n* = 262)	ICU survivors (*n* = 174)	ICU non-survivors (*n* = 88)	*p* value
Reason for ICU admission [*n* (%)]				0.42
Not related to cancer	174 (66.4)	120 (68.9)	54 (61.4)	
Related to anticancer drugs	49 (18.7)	29 (16.7)	20 (22.7)	
Related to cancer progression	39 (14.9)	25 (14.4)	14 (15.9)	
Reasons for ICU admission [*n* (%)]				<0.001
Sepsis	80 (30.5)	57 (32.8)	3 (26.1)	
Acute respiratory failure	74 (28.2)	47 (27)	27 (30.8)	
Coma or seizure	21 (8)	12 (6.9)	9 (10.2)	
Cardiorespiratory shock	18 (6.9)	1 (0.5)	17 (19.3)	
Hemorrhagic shock	16 (6.1)	14 (8.1)	2 (2.3)	
Acute kidney failure	14 (5.3)	10 (5.8)	4 (4.6)	
Acute heart failure	10 (3.8)	8 (4.6)	2 (2.3)	
Chronic bronchitis exacerbation	8 (3.1)	7 (4)	1 (1.1)	
Electrolytic disorders	6 (2.3)	5 (2.9)	1 (1.1)	
Hypovolemia	5 (1.9)	4 (2.3)	1 (1.1)	
Intentional overdose	1 (0.4)	1 (0.5)	0 (0)	
Post-surgery complications	1 (0.4)	1 (0.5)	0 (0)	
SAPS 2 score	61.9 ± 22.5	51.4 ± 13.0	82.0 ± 22.9	<0.001
Neutropenia	19 (7.3)	12 (6.9)	7 (8)	0.75
ICU stay and treatments				
Time in ICU [days, median (IQR)]	4 (2;7)	4 (2;7)0	4 (1;8)	0.26
Time in hospital [days, median (IQR)]	14 (5;25)	16 (8;27)	6 (2;16)	<0.001
Mechanical ventilation	135 (51.5)	54 (31)	81 (92.1)	<0.0001
Mechanical ventilation duration (days)	3.5 ± 6.3	2.2 ± 4.4	6.2 ± 8.3	<0.001
Noninvasive ventilation	25 (10.3)	20 (12.9)	5 (5.7)	0.08
Missing	19 (7.3)	19 (10.9)	0 (0)	
Inotropes	126 (48.3)	52 (30.1)	74 (84.1)	<0.0001
Missing	1 (0.4)	1 (0.6)	0 (0)	
Inotropes duration (days)	1.4 ± 2.4	0.8 ± 1.6	2.7 ± 3.1	<0.001
Dialysis	33 (12.6)	17 (9.8)	16 (18.2)	0.05
Missing	1 (0.4)	1 (0.6)	0 (0)	
Limitation of active therapies [*n* (%)]	46 (17.6)	11 (6.3)	35 (39.8)	<0.001

Data are mean ± SD or number of patients (%), expect for time in ICU and in hospital (median and interquartile [IQR]). Time in ICU and in hospital is median (IQR)

#### Outcomes

The ICU mortality rate in elderly solid tumor patients was 33.6% (*n* = 88), the in-hospital mortality rate was 43.9% (*n* = 115), while the 90-day mortality was 51.9% (*n* = 136, lost to follow-up *n* = 14). Out of the 174 patients with solid tumor who survived the ICU stay, 28 did not resume anticancer therapy because there was no treatment indication (i.e., localized tumor). Out of the 146 patients with advanced disease who theoretically had an indication for additional cancer therapy, 77 (52.7%) received treatment, 54 (37.0%) did not receive treatment, and 15 patients (10.3%) were lost to follow-up. Characteristics of the 146 ICU survivors with anti-tumoral treatment indication are presented in Additional file 1: Table S3.

### Prognostic factors analysis

#### Univariate analysis

Variables significantly associated with 90-day death were sex (*p* = 0.03), SAPS 2 score (*p* < 0.0001), mechanical ventilation (*p* < 0.0001), inotrope use (*p* < 0.0001), limitation of active therapies during ICU stay (*p* < 0.0001), primary tumor site (*p* = 0.005), leukocyte count (*p* < 0.0001), blood pH (*p* < 0.0001), lactate levels (*p* < 0.0001), aspartate aminotransferase level (*p* < 0.0001), alanine aminotransferase level (*p* = 0.01), bilirubin (*p* = 0.04) and albumin (*p* = 0.02) (Table 3). Performance status was not associated with 90-day mortality in univariate analysis (*p* = 0.07), as was metastatic status (*p* = 0.82).

**Table 3:** Independent predictors of 90-day mortality in elderly solid tumor patients (n = 262)

	Univariate		Multivariate	
	HR (95% CI)	*p* value	HR (95% CI)	*p* value
Age (HR for increase of 1 year)	0.98 (0.95–1.01)	0.16	0.99 (0.95–1.03)	0.57
Sex				
Male	1		1	
Female	0.66 (0.46–0.96)	0.03	0.75 (0.43–1.30)	0.30
Performance status				
0–1	1	0.07	1	0.17
2	1.30 (0.85–1.97)		1.58 (0.97–2.59)	
3–4	1.88 (1.10–3.23)		1.53 (0.77–3.05)	
Primary tumor site				
Genitourinary	1	0.005	1	0.01
Gastrointestinal	1.10 (0.65–1.86)		0.96 (0.49–1.89)	
Breast	0.82 (0.36–1.89)		0.38 (0.11–1.27)	
Lung	1.37 (0.83–2.26)		1.11 (0.59–2.08)	
Head and neck	2.60 (1.49–4.53)		2.69 (1.25–5.79)	
Other	0.75 (0.29–1.96)		0.80 (0.24–2.67)	
Metastatic status				
No	1		1	
Yes	0.96 (0.68–1.36)	0.82	1.61 (0.98–2.59)	0.06
Number of previous systemic anti-tumoral treatments	0.88 (0.73–1.07)	0.19	(–)	
Reason for ICU admission				
Not related to cancer	1 (–)	0.68	1	0.50
Related to anticancer drugs	1.07 (0.69–1.67)		0.87 (0.49–1.52)	
Related to cancer progression	1.23 (0.76–2.00)		1.23 (0.66–2.28)	
SAPS 2 score (HR for increase of 1 unit)	1.05 (1.04–1.06)	<0.0001	1.05 (1.03–1.06)	<0.0001
Life-support techniques				
Mechanical ventilation use	5.96 (3.91–9.10)	<0.0001	(–)	
Noninvasive ventilation use	0.81 (0.46–1.44)	0.48	(–)	
Inotropes use	3.68 (2.54–5.33)	<0.0001	(–)	
Dialysis use	1.28 (0.79–2.08)	0.32	(–)	
Limitation of active therapies during ICU stay	3.79 (2.59–5.56)	<0.0001	(–)	
Laboratory findings (HR for increase of 1 unit)				
Albumin	0.94 (0.89–0.99)	0.02	(–)	
Leukocytes	1.01 (1.01–1.02)	<0.0001	0.99 (0.97–1.01)	0.57
Glycemia	1.03 (1.00–1.05)	0.04	1.01 (0.98–1.06)	0.46
pH	0.04 (0.01–0.13)	<0.0001	0.85 (0.16–4.41)	0.84
Lactates	1.18 (1.13–1.22)	<0.0001	1.07 (0.99–1.15)	0.08
ASAT	1 (1–1.001)	<0.0001	1 (1–1)	0.58

*ASAT* aspartate aminotransferase, *CI* confidence interval, *HR* hazard ratio

#### Multivariate analysis

High SAPS 2 score (HR 1.05; 95% CI 1.03–1.06, *p* < 0.0001) and primary tumor site (*p* = 0.01) were significantly associated with 90-day death in patients with solid tumors admitted in ICU. When artificial life-support techniques were added to the model, SAPS 2, primary tumor site, metastatic status and lactates were predictive of 90-day death (Additional file 1: Table S4). The number and type of previous anticancer therapies and the performance status were not associated with death in multivariate analysis.

For the 146 patients with an indication for ongoing anticancer therapy, those with a gastrointestinal (*p* = 0.01) or lung (*p* = 0.02) tumor, or who had a performance status of 3 or 4 (*p* = 0.001) or who were admitted in ICU because of cancer progression (*p* = 0.04), were significantly less likely to have antineoplastic treatment restarted after ICU discharge (Table 4).

**Table 4:** Multivariate analysis of factors associated with definitive anticancer drug cessation after ICU discharge in survivors

	OR	95% CI	*p* value
Primary tumor site			
Genitourinary	1		
Gastrointestinal	8.70	1.61–47.15	0.01
Lung	6.92	1.28–37.38	0.02
Breast	1.41	0.10–20.17	0.80
Other	5.50	0.71–42.68	0.10
Performance status			
0 or 1	1		
2	2.26	0.82–7.60	0.14
3 or 4	14.96	2.71–78.68	0.001
Reason for ICU admission			
Not related to cancer	1		
Related to anticancer drugs	1.10	0.76–6.69	0.87
Related to cancer progression	4.19	1.03–17.14	0.04
Other			
LAT in ICU	191,146,845.57	0–Inf	0.99
Dialysis	3.19	0.67–15.28	0.14
High blood bilirubin level	1.00	0.99–1.02	0.48

*CI* confidence interval, *OR* odds ratio, *LAT* limitation of active therapies

## Discussion

This study provides interesting data about outcomes in elderly patients with active solid tumors admitted to the ICU, who comprised 14.3% of all elderly patients with an ICU admission over the study period (60.3% of these had metastatic disease). The presence of a solid tumor did not increase ICU mortality compared with patients who did not have a solid tumor, but elderly patients admitted to the ICU with active cancer were extremely critically ill. The majority of indications for ICU admission were not related to cancer. More than half of patients with an indication for ongoing cancer therapy had treatment resumed after their ICU stay. The 90-day mortality was 51.9%. We identified high SAPS 2 score and primary tumor site as being significantly associated with death at day 90 on multivariate analysis, whereas previous cancer therapies and performance status were not.

In our study, the ICU mortality rate for critically ill elderly patients with solid tumors was 33.6%, comparable to that in similar patients without cancer. Previous studies assessing the outcome of cancer patients in the ICU reported a wide range of mortality rates, from 24 to 75% [9, 11–15]. This wide range can be explained by the marked heterogeneity in patient case mix: medical and surgical patients; solid and hematological cancer patients; allogeneic and autologous bone marrow transplant recipients; and patients with and without early limitation of active therapies [5, 16]. In many studies, hematological cancer, medical admissions and limitation of active therapies were associated with worse prognosis [16]. In our study, we focused on patients with solid cancers and we excluded those with early limitation of active therapies.

To date, few studies in this setting have focused on elderly patients. In a registry of 1134 patients aged >65 years with advanced lung cancer admitted to ICUs, the in-hospital mortality was 33% [8], almost identical to the rate in our study. In the registry cohort, age was not associated with mortality, and this was also the case in multivariate analyses from other studies assessing predictors of death in critically ill patients admitting to the ICU with solid cancers [11, 12, 15–17].

In our cohort, patient prognosis after ICU admission appeared to be associated more with the severity of clinical condition at ICU admission than with the presence of cancer and age, as in most other previous studies [8, 9, 11–17]. In the Sepsis Occurrence in Acutely Ill Patients (SOAP) study, patients with solid tumors had similar survival to patients without cancer, as was the case in our analysis, although the SOAP study included patients aged <65 years [18]. Also in the SOAP study, hospital mortality increased as the number of organs failing increased, and more than 75% of the subset of patients with more than three failing organs died compared with about 50% of patients without cancer (*p* = 0.01). In a multicenter study conducted in Brazil (*n* = 717), higher Sequential Organ Failure Assessment scores were found to be associated with increased hospital mortality on multivariate analysis (odds ratio [OR] 1.25; 95% confidence interval [CI] 1.17–1.34) [11].

We found that primary cancer type was associated with 90-day mortality whereas number of previous cancer treatments was not. This is an area of debate, with recent studies that assessed factors associated with ICU death in cancer patients regardless of age and cancer type showing an inconsistent link between ICU death and cancer [5, 9, 16, 19, 20]; some authors have found association between cancer progression, metastatic status and prognosis [9, 11, 15]. In a large multicenter study including 717 patients (667 with solid tumors and 50 with hematological malignancies), active recurrent or progressive malignancy was associated with increased hospital mortality in multivariate analysis [11, 17]. Another study among patients with solid tumors (*n* = 162) found that complete or partial remission of the underlying cancer decreased the risk of ICU mortality (OR 0.026, 95% CI 0.002–0.3; *p* = 0.004) [15]. Our data showed that ICU admission related to tumor progression was not associated with mortality, but was associated with less resumption of anticancer treatment. However, we did exclude patients with early limitation of active therapies, and most admissions were not related to cancer progression. It has previously been shown that some specific causes of admission due to progression of solid tumors are associated with very poor prognosis [21, 22]. As a result, the elderly patients admitted to the ICU in this study might represent a selected group, and we did not assess patients not referred to the ICU by the oncologist or refused by the intensivist.

There is a relative lack of data about links between long-term outcome and anticancer therapy resumption [8, 9]. In our study, the number of patients able to resume antineoplastic therapy was encouraging (52.7%). This rate is high compared to the small amount of existing data. In one study of 1134 patients aged >65 years with active advanced lung cancer, only 19% resumed anticancer therapy after ICU discharge [8]. Another study about critically ill lung cancer patients reported a therapy resumption rate of 37% [9]. Possible reasons for the lower rates in the other studies could be the advanced stage of lung cancer, the high proportion of cancer-related complications and low number of therapeutic options in those patients. In addition, new targeted therapies in oncology and the fact that our cohort included other tumor types could have contributed to the different results between studies. It has been previously reported that there is a link between poor performance status and no resumption of anticancer treatment after an ICU stay [9]. Similarly, we found that gastrointestinal and lung cancer or patient admitted to the ICU because of tumor progression, or with poor performance status were prognostic factors for no anticancer therapy after ICU discharge. One explanation for this could be that patients with tumor progression may be more weakened by an ICU stay, and thus, further cancer therapy might be deemed too intense for them.

This study had several strengths. It is one of the largest cohorts in which long-term mortality of elderly patients with active cancer admitted to the ICU has been determined. Moreover, only patients with solid tumors were included, eliminating high-risk populations (e.g., patients with hematological malignancies. Another strength was the collection of data about tumor and metastatic sites, as well as the number and type of treatments received prior to ICU admission. Furthermore, we include data on resumption of anticancer therapy after ICU discharge, something that has only been reported in a few previous studies and is clinically relevant because it could play a part in assessing whether or not to admit a patient with cancer to the ICU.

Our study also has some limitations that need to be taken into account when interpreting the data. The study was conducted in a single center and had a retrospective design. Also, there were some database limitations, including missing data on biological and clinical variables (e.g., albumin level or performance status) meaning that calculations on the prognostic value of these variables may be affected. Furthermore, our results are only applicable to a subset of similar patients due to selection bias; patients too unwell to be admitted to the ICU were not included, as indicated by the fact that only 15% of our patients were in the ICU because of cancer progression. As a result, the study population had a relatively better general condition with fewer comorbidities, which may explain the good outcomes observed.

## Conclusions

Our study results showed that elderly patients (≥65 years) with solid tumor admitted to the ICU had the same mortality rate as similar elderly patients without cancer. In addition, more than half of the elderly cancer patients discharged from the ICU were able to resume anticancer treatment. Factors associated with ICU mortality were related to the patients’ status at ICU admission and not to the presence of metastatic disease or the number of previous anticancer treatments. These findings suggest that early admission of elderly cancer patients to the ICU is appropriate, when necessary.

## Additional file

**Additional file 1: Table S1.** Main characteristics of the whole population. **Table S2**. Biological data at admission in cancer patients (*n* = 262). **Table S3**. Characteristics of ICU survivors with anti tumoral treatment indication according to cessation/resumption of anti cancer drugs after ICU discharge. **Table S4**. Independent predictors of 90-days mortality (multivariate analysis including life supporting therapies).

## Abbreviations

95% CI: 95% confidence interval
HR: hazard ratio
ICU: intensive care unit
LATA: limitation of active therapies
SOAP: Sepsis Occurrence in Acutely Ill Patients
