# Supplementary material for: Outcomes in elderly patients admitted to the intensive care unit with solid tumors
Source: Ann Intensive Care. 2017 Mar 6;7:26. doi: 10.1186/s13613-017-0250-0 (PMC5339259; doi:10.1186/s13613-017-0250-0)
Supplement: Supplementary file 1 — Additional file 1: Table S1. Main characteristics of the whole population. Table S2. Biological data at admission in cancer patients (n = 262). Table S3. Characteristics of ICU survivors with anti tumoral treatment indication according to cessation/resumption of anti cancer drugs after ICU discharge. Table S4. Independent predictors of 90-days mortality (multivariate analysis including life supporting therapies). [file 13613_2017_250_MOESM1_ESM.docx]

|  | **Whole population**  **(n=2327)** | **Cancer population**  **(n=262)** | **Non cancer population**  **(n=1933)** | **P value** |
| --- | --- | --- | --- | --- |
| **Age, years** | 77.1 ± 8.1 | 75.2 ± 6.7 | 79 ± 8.2 | <0.0001 |
| **Sex male** | 1311 (56.3) | 162 (61.8) | 1101 (56.9) | 0.13 |
| **SAPS 2** | 59.4 ± 22.8 | 61.9 ± 22.5 | 56.9 ± 22.4 | <0.0001 |
| **Life-support therapies** |  |  |  |  |
| Mechanical ventilation | 1268 (54.5) | 135 (51.5) | 1133 (58.6) | 0.03 |
| Non invasive ventilation | 164 (7.0) | 25 (9.5) | 139 (7.2) | 0.17 |
| Inotrope use | 989 (42.5) | 126 (48.1) | 863 (44.6) | 0.29 |
| Dialysis | 530 (22.8) | 33 (12.6) | 497 (25.7) | <0.0001 |
| **ICU Death** | 758 (32.5) | 88 (33.6) | 633 (32.7) | 0.78 |

The values are mean ± SD or n (%)

**Table S1. Main characteristics of the whole population**

|  |  | **Whole population** | **ICU survivors** | **ICU non survivors** | **p-value** |
| --- | --- | --- | --- | --- | --- |
| **Mean (SD)** |  | **(N=262)** | **(N=174)** | **(N=88)** |  |
| **Albumine (g/L)** |  | 23.7 (6.2) | 24.3 (6.2) | 21.9 (6.0) | 0.12 |
|  | Missing | 169 | 102 | 67 |  |
| **Leucocytes (G/L)** |  | 14.3 (23.9) | 12.7 (10.0) | 17.5 (39.1) | 0.27 |
|  | Missing | 2 | 0 | 2 |  |
| **Lymphocytes (G/L)** |  | 2.4 (23.1) | 0.9 (0.9) | 5.4 (39.5) | 0.33 |
|  | Missing | 42 | 29 | 13 |  |
| **Platelets (G/L)** |  | 244 (294) | 253 (341) | 228(161) | 0.42 |
|  | Missing | 2 | 0 | 2 |  |
| **C-reactive protein (mg/L)** |  | 125.5 (117.3) | 118.3 (115.1) | 145.2 (122.3) | 0.20 |
|  | Missing | 93 | 50 | 43 |  |
| **Glycemia (mmol/L)** |  | 8.9 (5.8) | 8.6 (4.1) | 9.5 (8.3) | 0.40 |
|  | Missing | 8 | 1 | 7 |  |
| **Blood pH** |  | 7.30 (0.15) | 7.37 (0.11) | 7.28 (0.20) | <0.001 |
|  | Missing | 4 | 2 | 2 |  |
| **Lactates (mmol/L)** |  | 3.6 (4.2) | 2.6 (2.7) | 5.6 (5.6) | <0.001 |
|  | Missing | 17 | 12 | 5 |  |
| **ASAT (UI/L)** |  | 149 (516) | 80 (217) | 282 (818) | 0.03 |
|  | Missing | 10 | 8 | 2 |  |
| **ALAT (UI/L)** |  | 90 (334) | 54 (133) | 159 (535) | 0.08 |
|  | Missing | 10 | 8 | 2 |  |
| **Bilirubin (μmol/L)** |  | 22.4 (42.8) | 19.12 (30.1) | 28.6 (59.9) | 0.17 |
|  | Missing | 10 | 8 | 2 |  |
| **Serum creatinin (μmol/L)** |  | 197.2 (240.7) | 201.6 (265.4) | 188.3 (181.6) | 0.64 |
|  | Missing | 2 | 0 | 2 |  |
| **Uremia (mmol/L)** |  | 13.9 (14.4) | 13.4 (15.7) | 15 (11.3) | 0.88 |
|  | MIssing | 5 | 3 | 2 |  |

The values are mean ± SD

*ASAT :Aspartate aminotransferase, ALAT : Alanine aminotransferase*

**Table S2 :** biological data at admission in cancer patients (n=262)

|  |  | **ICU survivors with anti tumoral treatment indication** | **Anti tumoral treatment cessation after ICU discharge** | **Anti tumoral treatment resumption after ICU** | **Lost to follow up** | **p-value** |
| --- | --- | --- | --- | --- | --- | --- |
|  |  | **(N=146)** | **(N=54)** | **(N=77)** | **(N=15)** |  |
| **Sex** | Male | 82 (56.2%) | 29 (53.7%) | 46 (59.7%) | 7 (46.7%) | 0.58 |
|  | Female | 64 (43.8%) | 25 (46.3%) | 31 (40.3%) | 8 (53.3%) |  |
| **Performance status** | 0 or 1 | 50 (43.5%) | 10 (25.6%) | 37 (55.2%) | 3 (33.3%) | 0.01 |
|  | 2 | 48 (41.7%) | 18 (46.2%) | 25 (37.3%) | 5 (55.6%) |  |
|  | 3 or 4 | 17 (14.8%) | 11 (28.2%) | 5 (7.5%) | 1 (11.1%) |  |
|  | Missing | 31 | 15 | 10 | 6 |  |
| **Primary tumour site** | Breast | 11 (7.5%) | 1 (1.9%) | 9 (11.8%) | 1 (6.7%) | --- |
|  | Gastrointestinal | 43 (29.5%) | 22 (40.7%) | 18 (23.4%) | 3 (20%) |  |
|  | Genitourinary | 32 (21.9%) | 9 (16.7%) | 20 (25.9%) | 3 (20%) |  |
|  | Lung | 40 (27.4%) | 14 (25.9%) | 20 (25.9%) | 6 (40%) |  |
|  | Other | 20 (13.7%) | 8 (14.8%) | 10 (13.0%) | 2 (13.3%) |  |
| **Reason for ICU admission** | Not related to cancer | 95 (65.1%) | 34 (63%) | 52 (67.5%) | 9 (60%) | 0.40 |
|  | Related to anti cancer drugs | 26 (17.8%) | 8 (14.8%) | 16 (20.8%) | 2 (13.3%) |  |
|  | Related to cancer progression | 25 (17.1%) | 12 (22.2%) | 9 (11.7%) | 4 (26.7%) |  |
| **Inotrope use** | No | 105 (71.9%) | 39 (72.2%) | 55 (71.4%) | 11 (73.3%) | 1 |
|  | Yes | 41 (28.1%) | 15 (27.8%) | 22 (28.6%) | 4 (26.7%) |  |
| **Uremia** |  | 12.9 (14.6) | 11.3 (10.9) | 14.5 (17.3) | 10.6 (10.1) | 0.39 |
|  | Missing | 3 | 0 | 2 | 1 |  |

The values are n (%)

**Table S3 :** Characteristics of ICU survivors with anti tumoral treatment indication according to cessation/resumption of anti cancer drugs after ICU discharge

|  |  | **HR** | **95%CI** | **p-value** |
| --- | --- | --- | --- | --- |
| **Age** |  | 0.99 | 0.95-1.03 | 0.62 |
| **Sex** | Male | 1 |  |  |
|  | Female | 0.75 | 0.42-1.33 | 0.33 |
| **Primary tumor site** | Genitourinary | 1 |  | 0.004 |
|  | Gastrointestinal | 0.81 | 0.41-1.62 |  |
|  | Lung | 0.68 | 0.34-1.35 |  |
|  | Breast | 0.33 | 0.09-1.12 |  |
|  | Head and Neck | 2.41 | 1.08-5.37 |  |
|  | Other | 1.19 | 0.35-4.07 |  |
| **Metastatic status** | No metastasis | 1 |  |  |
|  | Metastatic | 2.15 | 1.30-3.54 | 0.003 |
| **Reason for ICU admission** | Not related to cancer | 1 |  | 0.96 |
|  | Related to anti cancer drugs | 0.92 | 0.52-1.65 |  |
|  | Related to cancer progression | 0.98 | 0.52-1.85 |  |
| **Performance status** | 0 or 1 | 1 |  | 0.24 |
|  | 2 | 1.50 | 0.91-2.47 |  |
|  | 3 or 4 | 1.09 | 0.54-2.19 |  |
| **SAPS 2 score** |  | 1.03 | 1.01-1.04 | 0.001 |
| **Life supporting therapies** | |  |  |  |
|  | Mechanical ventilation | 5.25 | 2.69-10.25 | <0.001 |
|  | Inotropes | 1.30 | 0.76-2.21 | 0.34 |
| **Laboratory finding** |  |  |  |  |
|  | Blood pH | 1.35 | 0.28-6.54 | 0.71 |
|  | Lactates | 1.12 | 1.04-1.22 | 0.004 |
|  | ASAT | 1 | 1-1 | 0.78 |
|  | Leucocyte count | 1 | 0.97-1.02 | 0.91 |
|  | Glycemia | 0.99 | 0.95-1.04 | 0.79 |

*ASAT :Aspartate aminotransferase,*

**Table S4 :** Independent predictors of 90-days mortality (multivariate analysis including life supporting therapies)
